# Chemical and Biological Characterization of the Anticancer Potency of *Salvia fruticosa* in a Model of Human Malignant Melanoma

**DOI:** 10.3390/plants10112472

**Published:** 2021-11-16

**Authors:** Sotiris Kyriakou, Venetia Tragkola, Michael Plioukas, Ioannis Anestopoulos, Paschalina S. Chatzopoulou, Eirini Sarrou, Dimitrios T. Trafalis, Maria V. Deligiorgi, Rodrigo Franco, Aglaia Pappa, Mihalis I. Panayiotidis

**Affiliations:** 1 Department of Cancer Genetics, Therapeutics & Ultrastructural Pathology, The Cyprus Institute of Neurology & Genetics, Ayios Dometios, Nicosia 2371, Cyprus; sotirisk@cing.ac.cy (S.K.); venetiat@cing.ac.cy (V.T.); ioannisa@cing.ac.cy (I.A.); 2 The Cyprus School of Molecular Medicine, Ayios Dometios, Nicosia 2371, Cyprus; 3 Department of Life & Health Sciences, School of Sciences & Engineering, University of Nicosia, Nicosia 2417, Cyprus; plioukas.m@unic.ac.cy; 4 Hellenic Agricultural Organization DEMETER, Institute of Breeding & Plant Genetic Resources, 57001 Thessaloniki, Greece; chatzopoulou@ipgrb.gr (P.S.C.); sarrou@ipgrb.gr (E.S.); 5 Laboratory of Pharmacology, Medical School, National & Kapodistrian University of Athens, 11527 Athens, Greece; dtrafal@med.uoa.gr (D.T.T.); mdeligiorgi@yahoo.com (M.V.D.); 6 Redox Biology Centre, University of Nebraska-Lincoln, Lincoln, NE 68583, USA; rodrigo.franco@unl.edu; 7 Department of Veterinary Medicine & Biomedical Sciences, University of Nebraska-Lincoln, Lincoln, NE 68583, USA; 8 Department of Molecular Biology & Genetics, Democritus University of Thrace, 68100 Alexandroupolis, Greece; apappa@mbg.duth.gr

**Keywords:** *Salvia fruticosa*, melanoma, oxidative stress, flavonoids, phenolics, apoptosis

## Abstract

Malignant melanoma is one of the most aggressive types of skin cancer with an increasing incidence worldwide. Thus, the development of innovative therapeutic approaches is of great importance. *Salvia fruticosa* (SF) is known for its anticancer properties and in this context, we aimed to investigate its potential anti-melanoma activity in an in vitro model of human malignant melanoma. Cytotoxicity was assessed through a colorimetric-based sulforhodamine-B (SRB) assay in primary malignant melanoma (A375), non-malignant melanoma epidermoid carcinoma (A431) and non-tumorigenic melanocyte neighbouring keratinocyte (HaCaT) cells. Among eight (8) different fractions of *S. fruticosa* extracts (SF1-SF8) tested, SF3 was found to possess significant cytotoxic activity against A375 cells, while A431 and HaCaT cells remained relatively resistant or exerted no cytotoxicity, respectively. In addition, the total phenolic (Folin–Ciocalteu assay) and total flavonoid content of SF extracts was estimated, whereas the antioxidant capacity was measured via the inhibition of *tert*-butyl hydroperoxide-induced lipid peroxidation and protein oxidation levels. Finally, apoptotic cell death was assessed by utilizing a commercially available kit for the activation of caspases - 3, - 8 and - 9. In conclusion, the anti-melanoma properties of SF3 involve the induction of both extrinsic and intrinsic apoptotic pathway(s), as evidenced by the increased activity levels of caspases - 8, and - 9, respectively.

## 1. Introduction

Skin cancer is one of the most frequent types of cancer, with increasing rates of incidence and mortality worldwide [1,2,3,4]. One of the most aggressive and deadly forms of skin cancer is malignant melanoma (MM) as it accounts for approximately 80% of all types of skin cancer-related deaths [1,3,5,6]. Current therapeutic schemes include surgical excision (in early stages), as well as chemotherapy, immunotherapy and targeted therapy (in advanced and metastatic stages). However, mortality rates remain high despite the development and improvement of therapeutic approaches. To this end, chemoprevention, utilizing synthetic and/or naturally derived agents, has emerged as a promising and alternative approach against cancer management, including MM [7,8,9].

Salvia species belong to the *Lamiaceae* family, which consists of 180 genera and approximately 3500 species [10,11]. *S. fruticosa (SF)* is an endemic plant mainly found in the Mediterranean and Irano-Turanian regions. It is also known as Greek sage, and has been reported to exert significant antioxidant and anticancer activity, possibly due to the presence of phenolic compounds (e.g., Rosmarinic acid; RA) that are well known for their antioxidant and antitumor effects [11,12,13,14]. In melanoma, RA was found to regulate different signalling pathways, including PKA/CREB/MITF, while also acts as a free radical scavenger and detoxification enzyme modifier [13,15]. Carnosic acid (CA) is another important diterpene phenolic compound, found in *S. fruticosa,* also known for its antioxidant and anti-inflammatory properties. Specifically, the treatment of melanoma cell lines with CA resulted in: (i) induction of apoptotic cell death; (ii) inhibition of epithelial-to-mesenchymal transition (EMT) and metastasis; as well as (iii) improvement of the cytotoxicity profile of several anticancer drugs [16,17].

Previous in vitro studies, conducted in a variety of cancer cell lines, revealed the beneficial effects of *S. fruticosa* extracts. For instance, Xavier et al. (2009) reported an anti-proliferative and pro-apoptotic activity of *S. fruticosa* in HCT-15 colorectal cancer cells while its anticancer activity was mediated through the inhibition of the Kirsten rat sarcoma virus (*KRAS*) gene and the reduction in reactive oxygen species (ROS) generation levels [12]. Similarly, a study conducted by Altay et al. (2017) indicated the anti-proliferative effect of *S. fruticosa* in HT-29 colorectal cancer cells primarily through its antioxidant capacity [15]. Moreover, *S. fruticosa* extracts were reported to possess significant anti-proliferative activity against melanoma cells, an effect associated with the G2/M phase of cell cycle growth arrest [16]. Finally, among the health-beneficial effects of *S. fruticosa* is its neuroprotective capacity against the formation of amyloid-beta plaques and subsequently, its protective potential against neurodegenerative diseases such as Alzheimer’s disease. Such activity was mainly attributed to its high content of antioxidant compounds including flavonoids, phenolics and terpenoids. Overall, the above data indicate that *S. fruticosa* plants contain promising phytochemicals, with notable biological activity and health-promoting capacity against a wide range of human diseases including cancer [18].

The purpose of the present study was to characterize the anticancer activity of different *S. fruticosa* extracts in an in vitro model of human MM consisting of primary malignant melanoma (A375), non-malignant melanoma epidermoid carcinoma (A431) and non-tumorigenic melanocyte-neighbouring keratinocyte (HaCaT) cells. This in vitro model allowed us to determine the cytotoxicity and safety profile of *S. fruticosa* between melanoma (A375) and non-melanoma (A431) carcinoma, as well as between tumorigenic (A375 and A431) and non-tumorigenic (HaCaT) cell lines, respectively. Such attempts can be of utmost importance in developing anticancer strategies for the clinical management of MM.

## 2. Results

### 2.1. S. fruticosa Extracts Induce Cytotoxicity in A375 Cells

**Figure 1:**
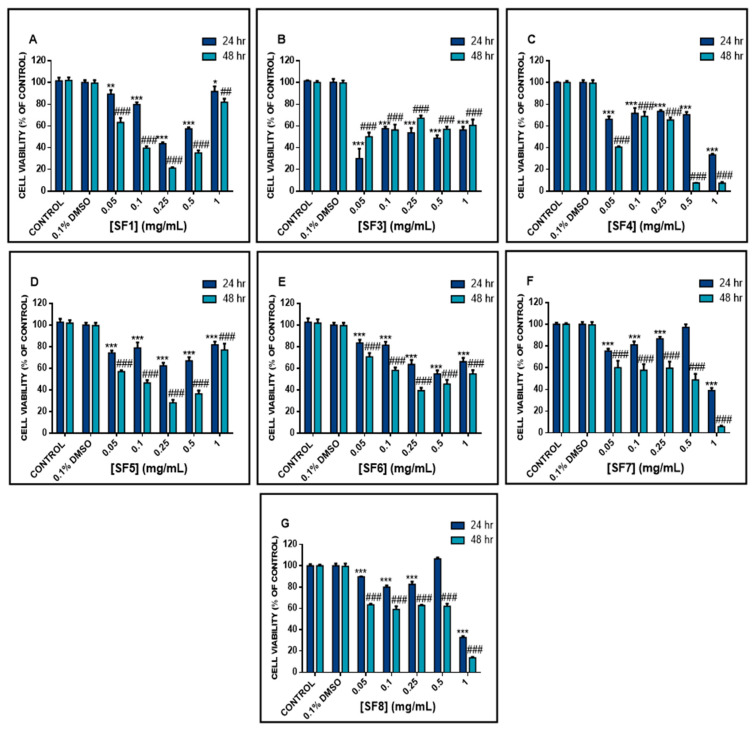
Cytotoxicity effect of various *S. fruticosa* extract fractions (SFs) in A375 cells. Cells were exposed to a range of 0.05–1 mg/mL concentrations of (**A**) SF1, (**B**) SF3, (**C**) SF4, (**D**) SF5, (**E**) SF6, (**F**) SF7 and (**G**) SF8 for 24 and 48 h. Data were expressed as mean values ± SD of 5 replicates from three independent experiments. Asterisk (*) denotes statistical significance when compared to their respective DMSO (0.1%) control at *p*  <  0.05. ** and ^##^ denote statistical significance at *p*  <  0.01, whereas *** and ^###^ denote statistical significance at *p*  <  0.001.

The cytotoxic capacity of *S. fruticosa* in A375 cells was evaluated, using the SRB assay, under increasing concentrations (0.05 mg/mL, 0.1 mg/mL, 0.25 mg/mL, 0.5 mg/mL and 1 mg/mL) of all extracts at 24 and 48 h of exposure. According to our data, all extracts exhibited a time-dependent reduction in cell viability (Figure 1), to a variable extent, as evidenced by their respective EC_50_ values (Table 1). Specifically, the SF4, SF7 and SF8 extract fractions induced a gradual decrease in cell viability (at both time points) followed by a sharp decline at 1 mg/mL concentration. Their corresponding EC_50_ values were 0.22, 0.56 and 0.88 mg/mL respectively. However, the SF1, SF5 and SF6 extract fractions were also shown to gradually decrease cell viability, at both time points, in all concentrations up to 0.5 mg/mL, followed by a marked increase (at 1 mg/mL) in cell viability values. Their respective effective concentration (EC_50_) values were calculated to be 0.82, 0.57 and 0.17 mg/mL, respectively. The most prominent cytotoxic response was observed with the methanolic extract fraction (SF3) where there was a marked and non-gradual reduction in cell viability levels in a manner independent of increasing concentrations and incubation periods of exposure. This was also evidenced by SF3 showing the lowest EC_50_ value (0.048 mg/mL) when compared to the rest of tested extracts. Consequently, given the above cytotoxic profile for each extract, we focused specifically on the SF3 extract fraction (at its EC_50_ value of 0.05 mg/mL) as being the most potent in exerting an anticancer capacity. 

Finally, it is worth mentioning that although we had collected an SF2 extract fraction (by using dichloromethane as an extraction solvent), it was not possible to screen it in our MM model as it was found to be poorly soluble in either water or dimethyl sulfoxide (DMSO) or methanol (MeOH), due to the precipitation of insoluble crystals.

### 2.2. The Methanolic Extract Fraction (SF3) Exerts Either No Cytotoxicity or a Minimal One in A431 and HaCaT Cells, Respectively

**Figure 2:**
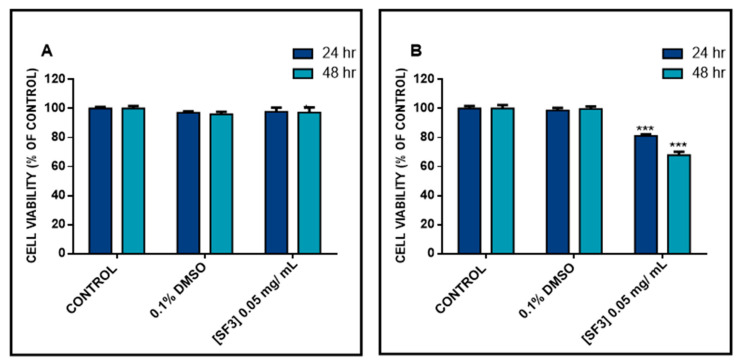
Cytotoxicity effect of the methanolic extract fraction (SF3) in HaCaT and A431 cells. (**A**) HaCaT and (**B**) A431 cells were exposed to SF3 at a concentration of 0.05 mg/mL, for 24 and 48 h. Data were expressed as mean values ± SD of 5 replicates from three independent experiments. Asterisks (***) demonstrate a statistical significance at *p*  <  0.001 relative to corresponding DMSO (0.1%) control.

In the next series of experiments, we examined whether the observed SF3-induced cytotoxicity was specific to A375 cells by utilizing a colorimetric SRB assay. To do so, non-melanoma (A431) and non-malignant keratinocyte (HaCaT) cells were treated with 0.05 mg/mL of SF3 for 24 and 48 h. The results revealed that cell viability was not affected in HaCaT-treated cells, at both time points of exposure, while, although a reduction in cell viability was observed in A431-treated cells, it was to a lesser extent when compared to A375 cells. Overall, our data support the notion that A375 are less resistant to the cytotoxic effect of the methanolic extract fraction (SF3) when compared to A431 and HaCaT cells, thereby indicating a melanoma-specific anticancer ability of this particular extract fraction of *S. fruticosa* (Figure 2).

### 2.3. The Methanolic Extract Fraction (SF3) Is a Rich Source of Phenolic and Flavonoid Compounds

Next, we sought to determine the total phenolic and flavonoid contents (TPC and TFC, respectively) of all *S. fruticosa* extract fractions in an attempt to identify the ones with major potential antioxidant capacity. Our results indicated that SF3 was the richest extract fraction in the context of having the highest TPC (319.188 μg of GAE/g of extract) and TFC (1054.66 μg of RE/g extract and 664.574 μg of CE/g extract) compared to the other fractions. On the contrary, the lowest TPC was observed in the SF8 fraction (46.38 μg of GAE/g of extract), while the lowest TFC was recorded in the SF2, SF5, and SF7 fractions (Table 2).

### 2.4. The Methanolic Extract Fraction (SF3) Exerts a Strong Antioxidant Capacity by Inhibiting Lipid and Protein Oxidation

**Figure 3:**
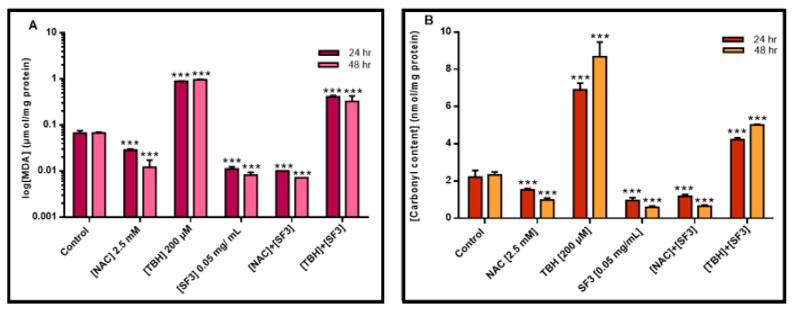
The effect of the methanolic extract fraction (SF3) in inhibiting lipid and protein oxidation in TBH-treated A375 cells. (**A**) Malondialdehyde (MDA) and (**B**) protein carbonyl contents upon treatments of A375 cells with 0.05 mg/mL of SF3 extracts, over 24 and 48 h of exposure, respectively. NAC (2.5 mM) and TBH (200 μΜ) were used as negative and positive controls, respectively. Data were expressed as mean values ± SD of 3 replicates from three independent experiments. Asterisks (***), denote statistical significance when compared to their respective control at *p*  <  0.001.

Next, we characterized the antioxidant capacity of the extract fraction (SF3) by determining its ability to inhibit lipid and protein oxidation measured as malondialdehyde (MDA) and protein carbonyl levels, respectively. Specifically, lipid peroxidation was measured by the TBARS assay while protein oxidation was measured by the use of dinitrophenylhydrazine, a compound that reacts with protein carbonyl groups. On both assays, *N*-acetyl cysteine (NAC) was used as a negative control (due to its antioxidant capacity) whereas *tert*-butyl hydroperoxide (TBH) was used as a positive one (due to its ability to induce lipid peroxidation). According to the results, it was evident that both NAC (2.5 mM) and SF3 (0.05 mg/mL) were effective in reducing, in a time-dependent manner and to a similar extent, MDA and carbonyl contents in A375 cells subjected to TBH (200 μΜ) (Figure 3A,B).

### 2.5. The Methanolic Extract Fraction (SF3) Induces Apoptosis in A375 Cells

**Figure 4:**
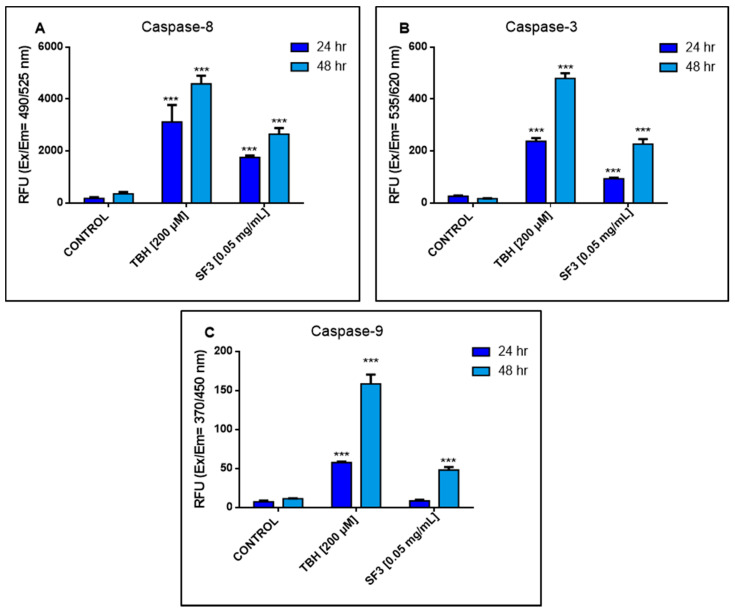
Determination of caspase activity in A375 cells following exposure to the methanolic extract fraction (SF3). (**A**) Caspase - 8, (**B**) Caspase - 3 and (**C**) Caspase - 9 activity levels were measured using fluorometric substrates for 24 and 48 h. Values are the means ± SD of 3 replicates from 3 independent experiments. Asterisks (***), *** at *p* <  0.001.

Finally, we investigated the type of SF3-induced cell death that occurred in A375 cells, with a particular focus on apoptosis. Following treatments of A375 cells with 0.05 mg/mL of SF3, for 24 and 48 h, the activity of Caspases - 3, - 8 and - 9 was measured using a commercial fluorometric multiplex assay kit. Our results indicated that the activity levels of Caspase - 8 were significantly increased (2000–3000 relative fluorescence units (RFU) on both time points of exposure) together with those of Caspase - 3 (although to a much lesser extent (100–250 RFU on both time points of exposure)). However, although the activity of Caspase - 9 was also increased, it was to a lesser extent when compared to that of Caspase - 8. Furthermore, the activity of Caspase - 9 was only minimally affected (10–50 RFU on both time points of exposure) (Figure 4).

## 3. Discussion

Melanoma is one of the most aggressive types of cancers with extremely low survival rates [19,20,21,22,23]. Previous studies have shown a beneficial effect of *S. fruticosa* against human breast and colorectal cancers [10,12,15,24,25]. Taken together, in the current study, we aimed to characterize the anticancer potential of various fractions of *S. fruticosa* extracts against malignant melanoma. To this end, we used seven (e.g., SF1, SF3, SF4, SF5, SF6, SF7 and SF8) *S. fruticosa* extract fractions obtained using different extraction solvents: petroleum ether, dichloromethane, methanol, diethyl ether, ethyl acetate, *n*-butanol and water. The differences in the polarity of each of the solvents utilized reflect the differences observed in the TPC, TFC and overall cytotoxicity profile data. In order to examine the cytotoxic effects of *S. fruticosa* extracts in melanoma, A375 cells were treated with these seven extract fractions under increasing concentrations ranging from 0.05 to 1 mg/mL. Interestingly, our results indicated that among all tested extract fractions, SF3 was the most potent in reducing cell viability levels of A375 cells. The corresponding EC_50_ value was calculated to be 0.048 mg/mL at 48 h post-exposure, and thus, was shown to be significantly lower when compared to the rest of the tested extracts. On the contrary, the SF1 and SF8 extract fractions were the least effective with corresponding EC_50_ values calculated to be 0.82 mg/mL and 0.88 mg/mL, respectively. In other words, they required 17–18-fold higher concentrations to reduce A375 cell viability to 50% when compared to SF3. Hence, SF3 was selected as the most cytotoxic extract for all subsequent experimental designs. Moreover, in another set of experiments, we aimed to examine the specificity of SF3-induced cytotoxicity, towards A375 cells only, by subjecting non-melanoma epidermoid carcinoma (A431) cells to the action of SF3. We showed that A431 cells were significantly more resistant to the cytotoxic action of this methanolic extract fraction, thereby suggesting a specific effect of SF3 in malignant melanoma cells. Obviously, this is largely speculation at this stage, and more elaborate experiments are needed in order to prove our initial hypothesis. Finally, we tested the cytotoxic effect of SF3 in non-malignant immortalized keratinocyte (HaCaT) cells on the basis that since they neighbour melanocytes, they could account for the determination of any cytotoxic side effects as a consequence of SF3’s action. In fact, when HaCaT cells were subjected to 0.048 mg/mL, over 48 h, no cytotoxicity was observed, thereby suggesting a safe profile of SF3 usage under these experimental conditions. In a relatively similar study, Koutsoulas et al. (2019) tested the methanolic extract fractions of *S. fruticosa* and *S. pomifera,* as well as carnosic acid itself in human melanoma (A375) and (Mel JuSo) cell lines. Their results revealed increased cytotoxicity on both cell lines with an IC_50_ value of 0.058 mg/mL in A375 cells [16]. Obviously, our results are in agreement with the finding of this study, as we reported an EC_50_ value of 0.048 mg/mL (compared to 0.058 mg/mL) for the methanolic extract fraction of *S. fruticosa* in A375 cells. Similar results have also been supported by other in vitro studies exploiting the cytotoxic effects of *S. fruticosa* in other forms of cancers (e.g., breast and colorectal), thereby proposing significant anticancer activity. For instance, in a study by Tundis et al. (2017), it was reported that the exposure of breast (MCF-7 and MB-231) and colorectal (Caco-2 and RKO) cancer cell lines to different concentrations of *S. fruticosa* resulted in a time- and concentration-dependent reduction in the viability levels of these cells [24]. Specifically, it was suggested that the cytotoxic effects of *S. fruticosa* are mainly attributed to the presence of carnosic acid [16], a strong antioxidant diterpene phenolic compound, with significant anti-neoplastic capacity against a variety of cancers including leukaemia, colorectal, brain and liver [16,26,27,28,29]. This, in turn, supports the notion of a synergistic effect between carnosic acid with other compounds such as phenols, flavonoids, pigments, sugars, etc., all of which are potentially present in the methanolic extract fraction of *S. fruticosa* and, thus, may contribute collectively to the observed anticancer capacity of this species. 

On another note, ROS are capable of attacking biological macromolecules such as lipids, proteins and DNA, thus resulting in the generation of lipid peroxidation, protein carbonylation and DNA oxidation by-products, all of which can ultimately lead to mutagenesis. For this reason, the presence of both enzymatic (e.g., Superoxide Dismutase (SOD), Glutathione Peroxidase (GPx), Catalase (CAT), etc.) and non-enzymatic antioxidants (e.g., ascorbic acid (vitamin C), α-tocopherol (vitamin E), glutathione (GSH), etc.), ensure the metabolism and elimination of excess ROS production [30,31,32,33]. However, when an imbalance between ROS production and anti-oxidant defence system occurs, it favours the accumulation of ROS, an unfavourable condition called oxidative stress that is closely related to carcinogenesis [34,35,36,37]. To these ends, increased ROS formation is highly associated with melanoma development, metabolism, immune response, melanin biosynthesis and metastasis [32,37,38,39]. Consequently, this suggests that naturally-derived phytochemicals, rich in antioxidant compounds, could potentially restore the imbalance favouring ROS generation back to a “physiological/redox state” by increasing the cellular metabolic activity of ROS and, thus, potentially inhibiting melanoma progression [30,31,32]. For instance, several studies have indicated the antioxidant capacity of various *Salvia* species, including *S. fruticosa*, attributed mostly to their phenolic and/or flavonoid compounds’ content [11,15,24,26,30,40,41], which can either donate hydroxyl groups to ROS and/or reactive nitrogen species (RNS) and/or chelate free metal ions that contribute to ROS formation and prevent their accumulation and, consequently, their detrimental effects [42,43,44]. Several studies conducted in melanoma and other skin cancers have revealed the significance of phenolic compounds as potential anticancer agents against both aggressive and non-aggressive types of skin cancer. For instance, cinnamic acid, a known phenolic compound, is capable of exerting a cytotoxic effect of HT-44 melanoma cells, while ferulic and caffeic acids were found to inhibit melanoma cell proliferation through down-regulation of PI3K/Akt signalling pathways [45,46,47,48]. Furthermore, a study published by Melo et al. (2018) utilizing phenolic compounds extracted from *Viscum album* tinctures, showed morphological changes and reduction in cell viability in B16F10 murine melanoma cell lines together with apoptotic activation [49]. In this context, in the present study, we measured the total phenolic and flavonoid contents of seven *S. fruticosa* extract fractions in order to investigate whether their observed cytotoxicity profiles are linked to the contents of these antioxidant compounds [30,50,51,52,53,54,55]. A high yield of total flavonoids and phenolics was expected in *S. fruticosa* extracts, as it has been already reported in previous studies [12,16,56,57]. Nonetheless, there was a discrepancy in the content between each of the tested extract fractions. More specifically, according to Ververis et al. (2020), the method of extraction (e.g., polar versus semi-polar versus non-polar extraction solvents) strongly affects the amount of total phenolic and flavonoid contents and subsequently affects the impact that each fraction has on cells [18]. As expected, in our study, the methanolic extract fraction (SF3) had the highest content on both phenolics and flavonoids, followed by SF4 and SF6, while the lowest content was observed in SF8 (for total phenolics) and in SF2 and SF7 (for total flavonoids). Furthermore, the antioxidant capacity of *S. fruticosa* was also evaluated through measuring its potential to inhibit protein carbonylation and lipid peroxidation contents. More specifically, increased intracellular ROS levels may result in direct damage of a protein’s amino acids and/or peroxidation of the lipid bilayer of cellular membranes, thus potentially leading to conformational changes associated with several diseases [58,59,60]. In this context, following incubations of A375 cells with TBH (a ROS-inducing agent), it was shown that both malondialdehyde (a lipid peroxidation marker) and carbonyl content (a protein oxidation marker) levels were significantly increased, as expected [61,62,63,64]. However, incubation of these cells with SF3 significantly reduced intracellular lipid and protein oxidation levels to a variable degree with a more notable degree of reduction regarding protein carbonyl content. 

Finally, in an attempt to further delineate the modes of the observed SF3-induced cytotoxicity, we employed a multiplex assay activity kit for caspases - 3, - 8 and - 9. According to our data, all three caspases were activated to a variable degree, suggesting the involvement of apoptotic induction as a response to the cytotoxic effect of SF3. According to different studies, exposure of breast and colon cancer cell lines to various *Salvia* species resulted in decreased cell viability levels through the induction of apoptotic cell death [25,65]. These observations are in agreement with ours not only in involving apoptotic induction as a mode of cell death but also in revealing the involvement of major caspases representing various modes of apoptosis. Specifically, we primarily showed the activation of the extrinsic apoptotic pathway, as evidenced by the significantly increased levels of activated Caspase - 8 [66,67,68,69]. Moreover, Caspase - 9 activation was found to be slightly increased, following SF3 exposure, indicating a minor involvement of the intrinsic apoptotic pathway [69,70,71,72]. In any case, we are reporting evidence of apoptotic induction primarily through the activation of Caspases of all three levels (Caspases - 3, - 8 and - 9), while the mode of such apoptotic induction (intrinsic versus extrinsic) remains largely speculative, at this stage, as more elaborate studies are needed to be performed in this direction.

## 4. Materials and Methods

### 4.1. Chemicals

Media and all cell culture related reagents were purchased from Biosera (Kansas City, MO, USA). Sulforhodamine-B (SRB) and trichloro-acetic acid (TCA) were purchased from Fluorochem (Glossop, UK), whereas Trisma Base, Aluminium trichloride and sodium acetate were purchased from Sigma-Aldrich (Merk) (St. Louis, MO, USA). TBARS and Protein Carbonyl Colorimetric Assay Kits were purchased from Cambridge Bioscience Ltd. (Cambridge, UK). The Folin–Ciocalteu Phenolic content assay kit was purchased from Bioquochem (Parque Tecnológico de Asturias, Llanera, Spain), whereas the Caspases - 3, - 8 and - 9 multiplex activity assay kit was purchased from Abcam (Cambridge, UK). All solvents were of UHPLC optima grade or better. 

### 4.2. Plant Material and Extract Preparation

The plant material, composed of the aerial parts of *S. fruticosa (SF)*, was collected during full flowering from selected *S. fruticosa* accessions, preserved in the experimental field of the Institute of Breeding & Plant Genetic Resources, (HAO DEMETER), Department of Medicinal & Aromatic Plants, Thessaloniki, Greece. The extraction and generation were carried out at the Department of Life & Health Sciences, School of Sciences & Engineering, University of Nicosia, Nicosia, Cyprus. Briefly, the air-dried SF aerial plant parts (leaves and flowers) were gradually extracted in a Soxhlet apparatus with petroleum ether, dichloromethane and methanol. The three obtained extracts (SF1, SF2, SF3) were concentrated to dryness under reduced pressure. The plant material was finally extracted with water, and the extract (SF4) was evaporated under vacuum to dryness. The methanolic extract was dissolved in boiling water, filtrated through a Whatman filter paper (pore size; 4.0–12 μm), and the filtrate was partitioned with three solvents of increasing polarity (diethyl ether, ethyl acetate, *n*-butanol). The organic layers of the above three solvents (SF5, SF6 and SF7) were concentrated to dryness and the remaining aqueous extract (SF8) was also collected. Stock solutions of each of the SF fractions were prepared in DMSO and kept at 4 °C, protected from light until use. 

### 4.3. Cell Lines

The human malignant melanoma (A375) cell line was purchased from American Type Culture Collection (ATCC—Manassas, VA, USA). The epidermoid carcinoma (A431) cell line was purchased from Deutsche Sammlung von Microorganismen und Zellkulturen (DSMZ—Braunschweig, Germany). HaCaT cells were kindly provided by Dr. Sharon Broby (Dermal Toxicology & Effects Group; Centre for Radiation, Chemical and Environmental Hazards; Public Health England, UK). All cell lines were maintained in a humidified chamber at 37 °C, under 5% CO_2_ atmosphere and according to the provider’s recommended culture conditions. All cell lines were cultured for 15–20 passages before new stocks were utilized. 

### 4.4. Determination of Cell Viability Levels

Cell viability levels were determined by utilizing the colorimetric SRB assay. Briefly, A375, A431, and HaCaT cells were seeded in 100 μL/well into 96-well plates and incubated overnight prior to exposure with each of the tested SF fractions. The density of A375 cells was 8000 and 4000 cells/well, whereas for A431 and HaCaT 10,000 and 5000 cells/well for 24 and 48 h of exposure, respectively. On the following day, cells were exposed to a range of concentrations (0.05–1.0 mg/mL) of each SF fraction over 24 and 48 h of exposure periods. Control cells were incubated with either complete medium only and/or DMSO (0.1%). At the indicated time points, cells were fixed by the addition of 25 μL of 50% *v*/*v* of ice-cold TCA solution and plates were incubated at 4 °C for 1 h. Then, the cells were washed 5x with distilled water and stained with 50 μL of 0.4% (*w*/*v*) SRB in 1% (*v*/*v*) acetic acid for 30 min at room temperature (RT). The unbound dye was removed through 5 washes with distilled water and the fixed/stained plates were air-dried overnight. Afterwards, the bound dye was solubilized by the addition of 100 μL of 10 mM Trizma base for at least 5 min. The absorbance was measured at 570 nm using a microplate reader (LT4500, Labtech, UK). Cell viability levels were calculated using the following Formula (1):

(1) $$\%\;\mathrm{Cell}\;\mathrm{viability}=\frac{{[\mathrm{sample}\;\mathrm{OD}}_{570}\;-\;\mathrm{blank}\;\left(\mathrm{media}\right){\mathrm{OD}}_{570}]}{{[\mathrm{mean}\;\mathrm{control}\;\mathrm{OD}}_{570}\;-\;\mathrm{blank}\;\left(\mathrm{media}\right){\mathrm{OD}}_{570}]}\;\times \;100$$

### 4.5. Determination of Total Phenolic Content (TPC)

For the determination of the Total Phenolic Content (TPC), SF fractions of equal concentrations were diluted (100×) with pre-warmed MeOH and syringe-filtered through a 0.45 μm pore filter membrane (Ministart, Sartorius stedim Biotech, Aubagne, France). Subsequently, TPC was quantitated by utilizing a commercial polyphenol quantification assay kit, based on the Folin–Ciocalteu method (Bioquochem, Spain), according to the manufacture’s protocol. The absorbance was monitored at 690 nm. The TPC was determined based on gallic acid calibration curve (linear range: 0–400 μg/mL; R^2^ > 0.991). The results were expressed as μg of gallic acid equivalents (GAE)/g of dry extract.

### 4.6. Determination of Total Flavonoid Content (TFC)

For the determination of Total Flavonoid Content (TFC), SF fractions of equal concentrations were diluted (100×) with pre-warmed MeOH and syringe-filtered through a 0.45 μm pore filter membrane (Ministart, Sartorius Stedim Biotech, Aubagne, France). Briefly, 20 μL of each fraction was further diluted with 60 μL of methanol and mixed with 10 μL of aluminium trichloride (10% aqueous solution), and 10 μL of sodium acetate (0.5 M aqueous solution). The resulting solutions were mixed by vortexing and allowed to stand in the dark at RT for 40 min. The absorbance was monitored at 415 nm. The TFC was determined based on both catechin (linear range: 0–100 μg/mL; R^2^ > 0.989) and rutin (linear range: 0–500 μg/mL; R^2^ > 0.995) calibration curve. The results were expressed as μg of catechin (CE) or μg of rutin (RE) equivalents/g of dry extract.

### 4.7. Determination of Malondialdehyde and Protein Carbonyl Contents

A375 cells were plated in 100 mm dishes (1.4 × 10^6^ and 0.7 × 10^6^ for 24 and 48 h, respectively) and cultured overnight. On the next day, cells were treated with either *N*-acetyl cysteine (NAC) (2.5 mM), *tert*-butyl hydroperoxide (TBH) (200 μΜ) or SF3 (0.05 mg/mL) for 24 and 48 h, respectively. After trypsinization, pellets were collected, re-suspended in PBS and sonicated. For the determination of the lipid peroxidation content, the whole suspension was further diluted with 4 mL of 4% *v*/*v* acetic acid solution containing 8% TBA and 0.1% SDS. The final mixture was heated at 95 °C for 1 h and centrifuged at 3000 rpm for 2 min. The TBARS Assay kit (Cambridge Bioscience Ltd., Cambridge, UK) was utilized for the determination of malondialdehyde (MDA) content according to the manufacture’s protocol. For the determination of protein carbonyl content, cells were trypsinized and pellets were collected, re-suspended in PBS (supplemented with 1 mM EDTA) and sonicated. The Protein Carbonyl Colorimetric Assay Kit (Cambridge Bioscience Ltd., Cambridge, UK) was utilized according to the manufacture’s protocol. 

### 4.8. Determination of Caspase Activity

Caspase activity was measured using the Caspases - 3, - 8 and - 9 multiplex assay (Abcam, UK). Briefly, A375 cells were plated in 96-well plates and, on the following day, were treated with the SF3 fraction (0.05 mg/mL). At the end of incubation, a caspase loading solution was prepared for each substrate through the addition of 50 μL of each caspase to 10 mL of Assay Buffer. Afterwards, 100 μL/well of caspase loading solution were added, without removal of the cell culture medium. The cells were incubated for 1 h at RT and fluorescence was measured using a fluorescence microplate reader (Synergy H1, Bio-Tek, VT, USS) at E*_ex_*/E*_em_* = 535/620 nm for caspase - 3, E*_ex_*/E*_em_* = 490/525 nm for caspase - 8 and E*_ex_*/E*_em_* = 370/450 nm for caspase - 9. The obtained results were expressed as Relative Fluorescence Units (RFUs).

### 4.9. Statistical Analysis

Data were expressed as mean values ± standard deviation (SD) and statistical analyses were performed by means of comparisons between control and treated cell populations. Statistical analyses were performed by one-way ANOVA with Tukey’s test for multiple comparisons using appropriate software (SPSS v.22). Finally, statistical significance was established at different levels (*p* < 0.05, *p* < 0.01 and *p* < 0.001).

## 5. Conclusions

Overall, our results provide evidence for the cytotoxic potential of *S. fruticosa* extracts against melanoma cells, an effect mainly attributed to its strong antioxidant activity and activation of apoptotic cell death. Future work can focus on characterizing the underlying molecular mechanism(s) that mediates apoptosis and/or other modes of cell death. To this end, the activation of anoikis cell death should not be excluded, since there is scientific evidence implicating carnosic acid (present in *S. fruticosa*) in the induction of anoikis in the B16F10 murine cell line [16]. Given the great need for developing alternative treatment options against melanoma, our results indicate that the methanolic fraction (SF3) of *S. fruticosa* can act as a potential anti-melanoma agent, thereby contributing to the development of novel pharmaceutical applications towards a more efficient clinical management of the disease. 

## Figures and Tables

**Table 1:** Half maximal effective concentrations (EC_50_) of various *S. fruticosa* extract fractions (SFs) against A375 cells, at 48 h post-exposure.

Fraction	Extraction Solvent	EC_50_ (mg/mL) (48 h)
**SF1**	Petroleum ether	0.82
**SF3**	Methanol	0.048
**SF4**	Water	0.22
**SF5**	Diethyl ether	0.57
**SF6**	Ethyl acetate	0.17
**SF7**	*n*-Butanol	0.56
**SF8**	Water	0.88

**Table 2:** Determination of total phenolic and flavonoid contents in various extract fractions of *S. fruticosa.* Data shown are mean values ± SD of at least three independent experiments.

	SF1	SF2	SF3	SF4	SF5	SF6	SF7	SF8
**TPC** **(μg GAE/ g of dry extract)**	136.26 ± 2.8	91.5 ± 5.9	319.19 ± 10.0	198.99 ± 28.7	110.01 ± 4.9	148.38 ± 3.8	82.13 ± 4.4	46.38 ± 5.7
**TFC** **(μg RE/ g of dry extract)**	788.33 ± 64.7	164.44 ± 6.0	1054.66 ± 58.2	685.66 ± 10.9	189.45 ± 3.2	187.97 ± 10.3	201.91 ± 1.2	583.59 ± 4.1
**TFC** **(μg CE/ g of dry extract)**	583.76 ± 12.7	104.08 ± 5.5	664.57 ± 15.2	235.32 ± 9.2	102.53 ± 5.9	175.9 ± 4.7	100.79 ± 7.9	226.88 ± 4.1

## Data Availability

Data is contained within the article.
